# Reciprocity

**Published:** 1899-08-19

**Authors:** 


### The Hospital

A Journal of

The Medical Sciences and Hospital Administration.

Vol. XXVI.—No. 673.] Edited by Sir Henry Burdett, K.C.B., and Solomon C. Smith, M.D., M.R.C.P. [August 19, 1899.

### RECIPROCITY.

In a report to the registered practitioners of England and Wales, Mr. Victor Horsley draws attention, among other things, to a point which has been far too much overlooked by those who profess anxiety to see reciprocity of medical practice established between this country and others, namely, that such an arrangement would render entirely nugatory the whole system of medical education and registration which has been established in this country. He says that during the last session the question came up again in reference to a sort of international bargain which was proposed by the Italian Ambassador, and concerning which the Executive Committee of the General Medical Council had expressed a desire to see the rights and privileges of medical practitioners in this country extended to all foreigners who could offer in return the privileges of practice in their own country; and it is, of course, easy to see that, if ever reciprocity were to be arranged on such terms, a back door to qualification would be opened such as would be very difficult of control. We think it high time, however, that this reciprocity bubble should be pricked. The question is discussed in many quarters as if the Italians were making a very reasonable request, and as if we were acting scurvily in raising any opposition to it. The truth is that the whole thing is a bogus demand, made because it is known that it cannot be granted, and with the special object of using the expected refusal as an excuse for action against the English medical men who now practise in Italy.

Everyone knows that if a medical man from abroad were to come to England and without acting as an apothecary, or assuming any medical title, or doing anything to imply that he was a registered medical practitioner, were to practise his profession among his compatriots, the law could not, or at any rate, would not, interfere with him. Such a state of the law is to be regretted, no doubt, but there it is; so that already foreigners in England possess greater freedom in the practice of their profession than Englishmen do in most foreign countries, for in neither France, nor Germany, nor Switzerland, would an Englishman, however highly qualified, be allowed to practise without passing an examination, and, in some cases, undergoing a course of study in the country in question. In the countries named this rule is strictly enforced, and even in Italy we believe the rule exists, so far as the letter of the law goes, but for reasons of policy which are easy to understand it has in that country been held in abeyance in regard to foreigners practising solely among their own countrymen. There is, then, already a practical reciprocity between England and Italy. Neither Government professes any anxiety to flood the other country with medical practitioners, but each asks that men of undoubted qualification and position shall be enabled to practise among their own countrymen, and this demand is met in England by the fact that the law does not interfere with practitioners so long as they do not wilfully and falsely take any title which implies that they are registered medical practitioners, and in Italy by an ordinance issued by the Government to the effect that the law shall not be put in force against such foreign practitioners as practise only among their own compatriots. Why, then, this demand for reciprocity? Well, hereby hangs a story which is not altogether unflattering to the position attained by some of the English physicians practising in Italy. It has, we believe, been found somewhat difficult to prevent well-to-do Italians, who like well-to-do people everywhere else, have much faith in the power of money, from going to the English doctors for advice, and their doing so has given great offence to the profession in Italy. And perhaps justly so. Their wrath, moreover, has been by no means lessened by the knowledge that their fellow countrymen were in many cases paying to these foreign intruders far higher fees than they would ever dream of offering for native talent. It was indeed an insult as well as an injury, and when some time ago a very high personage indeed commanded the attendance of an English physician it was felt that the thing could no longer be borne. For the last two or three years the medical profession in Italy has been unceasing in its efforts to stir up this question, and to obtain the literal enforcement of a law similar to those in operation in Germany and Switzerland. Needless to say, the hotel keepers do not see with them in this matter entirely eye to eye. In the end the compromise we have mentioned has come into being. But this is not enough for the native doctors. They know well enough how difficult it is to prevent rich people from going to whomsoever they choose in regard to such a matter as medical advice, and they want, whatever may be the cost to their country, to weed out the interlopers. They have the letter of the law behind them; all they want is a good excuse for demanding that it shall be enforced, and this they hope to get in the refusal of England—the anticipated and calculated upon refusal—to allow reciprocity of practice. Hence the demand. And that is the whole matter. But we in England, having gradually improved our system of registration to its present point, which surely is not too high, are not likely to open a back door to foreigners just to make things easy for a few gentlemen practising abroad. We need not fret, however. The English and American settlement in Italy is far too valuable an asset to be seriously interfered with, and we may be pretty sure that, if we do but let things alone, the Italians will somehow or another find a way. At the same time, we think that if English doctors are allowed to practise among their own countrymen in Italy they should place Italian patients out of bounds. It is the attendance on the natives that is the *casus belli*, and that ought to be given up.

